# Incursion of Novel Eurasian Low Pathogenicity Avian Influenza H5 Virus, Australia, 2023

**DOI:** 10.3201/eid3012.240919

**Published:** 2024-12

**Authors:** Michelle Wille, Victoria Grillo, Silvia Ban de Gouvea Pedroso, Natasha D. Brohier, Ivano Broz, Charlotte Burgoyne, Allison Crawley, Kelly Davies, Mark Ford, Joanne Grimsey, Nina Y.H. Kung, Jasmina M. Luczo, Cornelius Matereke, Peter T. Mee, Patrick Mileto, Matthew J. Neave, Megan Poon, Vittoria Stevens, Guy Weerasinghe, Sara Zufan, Ian G. Barr, Marcel Klaassen, Andrew C. Breed, Frank Y.K. Wong

**Affiliations:** Centre for Pathogen Genomics, The University of Melbourne, Melbourne, Victoria, Australia (M. Wille, S. Zufan); Peter Doherty Institute for Infection and Immunity, Melbourne (M. Wille, I.G. Barr); Wildlife Health Australia, Canberra, Australian Capital Territory, Australia (V. Grillo, S.B. de Grouvea Pedroso); Department of Energy, Environment, and Climate Action, Bundoora, Victoria, Australia (N.D. Brohier, P.T. Mee); Australian Centre for Disease Preparedness, Geelong, Victoria, Australia (I. Broz, K. Davies, M. Ford, J. Grimsey, J.M. Luczo, P. Mileto, M.J. Neave, M. Poon, V. Stevens, F.Y.K. Wong); Northern Australia Quarantine Strategy, Department of Agriculture, Fisheries, and Forestry, Darwin, Northern Territory, Australia (C. Burgoyne, G. Weerasinghe); Department of Primary Industries and Regions, Glenside, South Australia, Australia (A. Crawley, C. Matereke); Department of Agriculture and Fisheries Coopers Plains, Queensland, Australia (N.Y.H. Kung); Deakin University, Geelong (M. Klassen); University of Queensland, Brisbane, Australia (A.C. Breed); Department of Agriculture, Fisheries, and Forestry, Canberra (A.C. Breed)

**Keywords:** avian influenza, highly pathogenic avian influenza, low pathogenicity avian influenza, virus, influenza, H5, Australia, respiratory infections

## Abstract

Australia is a sink for low pathogenicity avian influenza viruses, with isolated circulation occurring on the continent. We report the incursion of a Eurasian low pathogenicity avian influenza H5 virus into Australia. This report benefits surveillance and diagnostic work because of the risk and current absence of highly pathogenic avian influenza A(H5N1).

Australia is a sink for the diversity of avian influenza viruses, wherein viruses circulating in Eurasia and North America are occasionally introduced to the continent, followed by long-term, isolated circulation (*1*). Whereas a diversity of low pathogenicity avian influenza (LPAI) viruses exist, Oceania is the only continent where the goose/Guangdong lineage of highly pathogenic avian influenza (HPAI) A(H5N1) is absent (*2*). An HPAI incursion has never been detected in Oceania, although several HPAI outbreaks have occurred in poultry, all following evolution of endemic Australian lineage LPAI H7 viruses from the wild bird reservoir (*3*). Because of the risk to poultry and wild birds in Australia, a key aim of the National Avian Influenza in Wild Birds (NAIWB) surveillance program is the detection and characterization of circulating H5 and H7 viruses (*4*).

## The Study

In 2023, through both the NAIWB targeted surveillance (*4*) and passive surveillance, we detected 17 LPAI H5 viruses by quantitative PCR (qPCR) and recovered 8 genomes. The genomes comprised 7 complete or near-complete genomes and 1 partial genome, which included hemagglutinin (HA), nucleoprotein, neuraminidase (NA), matrix, and nonstructural protein (NS) segments. We recovered viral HA fragments from 2 additional detections that identified lineage only (Table). We collected, screened, and sequenced samples as described in previous publications (*1*). We deposited the sequences into GenBank (GenBank accession nos. PP947529–52 and PP922943–79).

**Table:** Genome constellations for each virus sequenced in the study of an incursion of novel Eurasian low pathogenicity avian influenza H5 virus, Australia, 2023*

Virus designation	Date	PB2	PB1	PA	HA	NP	NA	M	NS
A/chestnut teal/Victoria/23-01686-0034/2023 (H5N3)	2023 Apr 26	Aus	Aus (B)	Aus	H5–EUR-novel	Aus	N3–Aus	Aus (A)	A–Aus
A/Pacific black duck/Victoria/23-01686-0039/2023 (H5N3)	2023 Apr 26	Aus	Aus (A)	Aus	H5–EUR-novel	Aus	N3–Aus	Aus (A)	B–Aus
A/gray teal/Victoria/23-01688-0045/2023 (mixed)†	2023 Apr 26	Aus	Aus (A)	Aus	H5–EUR-novel	Aus	N3–Aus/ N7–Aus	Aus (A)	B–Aus
A/Pacific Black Duck/Victoria/17363/2023 (H5N3)	2023 Jun 22	Aus	Aus (A)	Aus	H5–EUR-novel	Aus‡	N3–Aus	Aus (A)	A–Aus
A/wild waterbird/Queensland/ P23–02457–48/2023 (H5N3)	2023 Jun 23	Aus	Aus (C)	Aus	H5–EUR-novel	Aus	N3–Aus	Aus (B)	A–Aus
A/wild waterbird/Queensland/ P23–02457–51/2023 (H5N3)	2023 Jun 23	Aus	Aus (C)	Aus	H5–EUR-novel	Aus	N3–Aus	Aus (B)	A–Aus
A/wild waterbird/South Australia/23–80999145–13/2023 (H5N9)	2023 Jul 31	Aus	Aus (C)	Aus	H5–EUR-novel	Aus‡	N9–Aus	Aus (A)	B–Aus
A/Radjah Shelduck/Northern Territory/ 20231282–03/2023 (H5N1)	2023 Oct 30	ND	ND	ND	H5–EUR-novel	Aus‡	N1–Aus	Aus (A)	A–Aus

*In cases where viruses fell into different Australian lineages we added an indicatory letter in parentheses to show the different lineages. In the case of NS, whether the virus fell into the A or B allele is indicated before the lineage name. (e.g., B–Aus for an Australian lineage in the B allele). Similarly, for the neuraminidase subtype information is incorporated (e.g., N1–Aus for an Australian lineage in the N1). Aus, established Australian clade; EUR-novel, novel incursion from Eurasian lineage; HA, hemagglutinin; M, matrix protein; ND, no sequence data available; NA, neuraminidase; NP, nucleoprotein; NS, nonstructural protein; PA, polymerase acidic; PB, polymerase basic. †A/gray teal/Victoria/23-01688-0045/2023 comprised H5 and H10, and N3 and N7. ‡Cases where viruses fell into the same Australian lineage but were not sister to each other.

**Figure 1:**
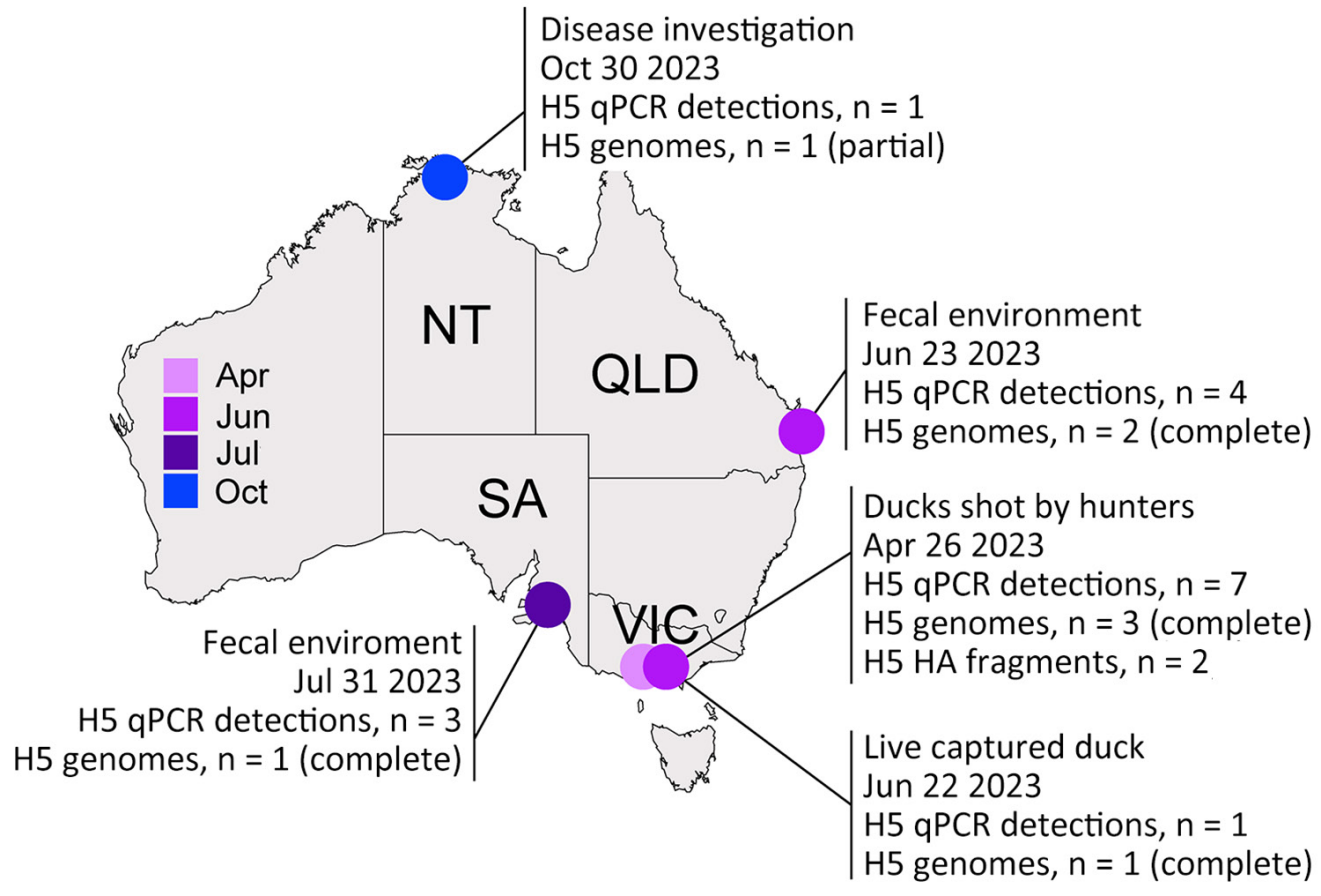
Locations of Eurasian lineage low pathogenic avian influenza H5 virus detections in Australia, 2023. Information is provided about the location, sample type, and date of H5 detections. NT, Northern Territory; QLD, Queensland; SA, South Australia; VIC, Victoria.

The HA segments of LPAI H5 viruses we sequenced were unrelated to LPAI H5 viruses previously characterized in Australia. The HA sequences had a PQRETR/GLF cleavage site, which contrasts with the PQKATR/GLF cleavage site found in all LPAI H5 viruses reported in Australia since 2005 (*1*). The detections we found all belonged to a single Eurasian-lineage LPAI H5 that was detected in 4 Australia jurisdictions over a 7-month period. We detected the first occurrence of the LPAI H5 in April 2023 (Figure 1) in Victoria from 3 hunter shot waterfowl: 1 Pacific black duck (*Anas superciliosa*), 1 gray teal (*Anas gracilis*), and 1 chestnut teal (*Anas castanea*). In June 2023, we detected another LPAI H5 occurrence in Victoria in a live-captured Pacific black duck and 2 LPAI H5 occurrences in Queensland from wild waterbird fecal environmental samples. We detected another LPAI H5 occurrence in July 2023 from wild waterbird fecal samples in South Australia. Finally, we detected 1 LPAI H5 virus in a wild duck in October 2023 through passive surveillance in the Northern Territory. Samples collected from live birds were done in accordance with Deakin University Animal Ethics (ethics approval no. B39-2019). Ethics approvals were not required for fecal environmental samples, samples collected from hunter-shot birds, or samples collected for disease investigations.

**Figure 2:**
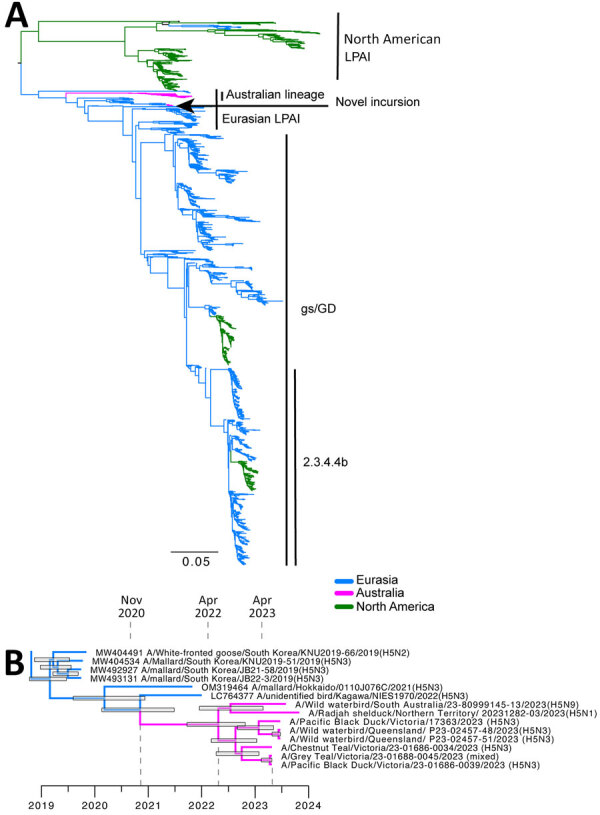
Phylogenetic analysis of Eurasian lineage LPAI H5 virus detected in Australia, 2023. A) Maximum-likelihood phylogenetic tree of all H5 sequences from Asia, North America, and Oceania since 2010. Major clades have been highlighted. The arrow indicates the novel lineage detection in Australia. The tree has been rooted between the North American and Eurasian lineages. Scale bar indicates number of substitutions per site. B) Cropped time-structured phylogenetic tree. Dashed lines indicate the date of divergence from GenBank reference sequences, the most recent common ancestor of all Australian sequences, and the date of first detection. Node bars comprise the 95% highest posterior density. Scale bar is time in years. gs/GD, goose/Guangdong lineage of highly pathogenic avian influenza A(H5N1); LPAI, low pathogenicity avian influenza.

We conducted BLASTn analysis (https://blast.ncbi.nlm.nih.gov) by using the National Center for Biotechnology Information nucleotide database. We conducted phylogenetic analysis of the HA sequences and found the sequenced viruses were most closely related (<98%) to LPAI H5 viruses detected during 2019–2022 in Japan and South Korea (Figures 1, 2). Those countries contribute most influenza genomes recovered from the East Asian–Australasian databases. Alignments and .xml files for all trees are available at https://github.com/michellewille2/Eurasian-LPAI-H5-incursion-to-Australia. We investigated the potential temporal incursion window in Australia by using time-structured phylogenetic analysis, as outlined in previous publications (*1*). The most recent common ancestor of the 8 complete HA sequences was from April 2022 (95% highest posterior density [HPD] September 2021–October 2022), almost a year before the first detection, but with a large HPD (Figure 1). To examine H5 activity in the previous year, we queried all H5 detections for 2022 reported to the NAIWB surveillance program and found 12 LPAI H5 viruses were detected by qPCR in wild bird samples (New South Wales, n = 3; Tasmania, n = 1; Victoria, n = 5; Western Australia, n = 3) (*5*). Of the viruses sequenced, all HA sequences fell into the previously described Australian LPAI H5 lineage that has been circulating since 2007, although it was likely present since the 1990s (*1*). Hence, cryptic circulation of the novel Eurasian lineage LPAI H5 in Australia may have occurred in locations or in avian hosts that are not included in NAIWB surveillance and therefore were not detected in 2022. Alternatively, incursion might have only occurred in early 2023, but rather than a single viral introduction, multiple viruses with closely related HA sequences were separately introduced. Unfortunately, the scarcity of available HA reference sequences from 2022 and 2023 from Asia because of limitations in wild bird surveillance or sequence deposition into GenBank hinders understanding of introduction dynamics.

We investigated the reassortment patterns in the segments to gain additional insight. All other gene segments fall into viral lineages already present in Australia (Table; Appendix Figure, Tables 1, 2). Genome constellations were partially conserved, and all viruses shared the same polymerase basic (PB) 1, polymerase acidic, nucleoprotein, and matrix segment lineages, although within-clade segments were not always 100% identical (Appendix Figure). We detected 3 different PB1 lineages and 2 different NS lineages (both the A and B alleles). We obtained full genomes of 4 viruses from wild birds in Victoria, 3 from the same sampling event. Of the full genomes obtained, 2 had identical genome constellations, although A/gray teal/Victoria/23-01688-0045/2023 was a mixed infection with H5, H10, N3 and N7 segments recovered. Co-sampled A/chestnut Teal/Victoria/23–01686-0034/2023(H5N3) was different in both the PB1 and NS sequence compared with the other 2 viruses. A/Pacific black duck/Victoria/17363/2023(H5N3), which was detected 2 months after the initial detections in Victoria, had a similar genome constellation to the other sequences from Victoria, despite having an HA sequence sister to those from Queensland (detected June 2023), rather than those from Victoria. A/wild waterbird/South Australia/23–80999145–13/2023(H5N9) had a different PB1 and NA sequence, and A/radjah shelduck/Northern Territory/20231282–03/2023(H5N1) also had a different NA subtype. The 2 genomes from Queensland shared the same PB1 sequence as A/wild waterbird/South Australia/23-80999145-13/2023(H5N9) but had unique M segments. A reasonable explanation for those results is a single introduction followed by a reassortment event incorporating 7 Australian-lineage segments. As the virus spread across Australia, additional reassortment events contributed to the genome constellation diversity (i.e., diversity in the PB1, NA, and NS segments).

## Conclusions

There are 3 implications of this viral incursion. First, the delayed detection highlights limitations in the national targeted surveillance program that, in combination with investigation of consequential wild bird illness and death events, is necessary to rapidly detect and respond to an incursion of goose/Guangdong HPAI H5N1 into Australia. Second, while the KATR cleavage site found in Australian-lineage viruses has not yet been associated with evolution of LPAI to HPAI, the RETR cleavage site in the novel Eurasian lineage incursion has been identified as a precursor to HPAI H5 viruses (*6*). This incursion may increase the risk for evolution of HPAI H5 in Australia. Finally, as part of the surveillance and diagnostic processes, H5 qPCRs are designed to rapidly differentiate LPAI H5 of Australian lineage from Eurasian lineage LPAI and HPAI. The presence of a novel LPAI lineage requires a diagnostic update. Understanding viral incursions is a global concern, as demonstrated by the numerous incursions and intercontinental spread of HPAI H5N1 clade 2.3.4.4b. Determining the patterns of viral incursion and spread and identifying the hosts involved is necessary for effective disease risk management and communication that supports disease preparedness and response.

Appendix Additional information about incursion of novel Eurasian low pathogenicity avian influenza H5 virus, Australia, 2023.

## References

[1] Wille M, Grillo V, Ban de Gouvea Pedroso S, Burgess GW, Crawley A, Dickason C, et al. Australia as a global sink for the genetic diversity of avian influenza A virus. PLoS Pathog. 2022;18:e1010150. 10.1371/journal.ppat.1010150 35536868 PMC9089890

[2] Wille M, Atkinson R, Barr IG, Burgoyne C, Bond AL, Boyle D, et al. Long-distance avian migrants fail to bring 2.3.4.4b HPAI H5N1 into Australia for a second year in a row. Influenza Other Respir Viruses. 2024;18:e13281. 10.1111/irv.13281 38556461 PMC10982072

[3] Scott A, Hernandez-Jover M, Groves P, Toribio JA. An overview of avian influenza in the context of the Australian commercial poultry industry. One Health. 2020;10:100139. 10.1016/j.onehlt.2020.100139 32490131 PMC7256052

[4] Grillo VL, Arzey KE, Hansbro PM, Hurt AC, Warner S, Bergfeld J, et al. Avian influenza in Australia: a summary of 5 years of wild bird surveillance. Aust Vet J. 2015;93:387–93. 10.1111/avj.12379 26503532

[5] National Avian Influenza Wild Bird Program. Wild bird news, issue 9. 2023 [cited 2024 Sep 12]. https://wildlifehealthaustralia.com.au/Resource-Centre/Surveillance-Reports

[6] Luczo JM, Stambas J, Durr PA, Michalski WP, Bingham J. Molecular pathogenesis of H5 highly pathogenic avian influenza: the role of the haemagglutinin cleavage site motif. Rev Med Virol. 2015;25:406–30. 10.1002/rmv.1846 26467906 PMC5057330

