# Supplementary material for: Incursion of Novel Eurasian Low Pathogenicity Avian Influenza H5 Virus, Australia, 2023
Source: Emerg Infect Dis. 2024 Dec;30(12):2620–4. doi: 10.3201/eid3012.240919 (PMC11616663; doi:10.3201/eid3012.240919)
Supplement: Appendix — Additional information about incursion of novel Eurasian low pathogenicity avian influenza H5 virus, Australia, 2023. [file 24-0919-Techapp-s1.pdf]

Article DOI: https://doi.org/10.3201/eid3012.240913

EID cannot ensure accessibility for supplementary materials supplied by authors. Readers who have difficulty accessing supplementary content should contact the authors for assistance.

Incursion of Novel Eurasian Low Pathogenicity Avian Influenza H5 Virus, Australia, 2023

Appendix.

Appendix Table 1. Top 5 Blast hits for neuraminidase segments against the NCBI nt database (September 2024)

NA Subtype	Designation	Top blast hit	% identity
N1	A/Radjah Shelduck/Northern Territory/ 20231282-03/2023(H5N1)	OL370361 A/wild waterbird/South Australia/19-9013889-61/2019(mixed)	98.01%
		OL370353 A/wild waterbird/South Australia/19-9013889-37/2019(N1)	98.01%
		OL371577 A/wild waterbird/South Australia/19-9013889-35/2019(H1N1)	97.94%
		OL371593 A/wild waterbird/South Australia/19-9013889-43/2019(H7N1)	97.94%
		OL370347 A/wild waterbird/South Australia/19-9013889-13/2019(N1)	97.87%
N3	A/Chestnut Teal/Victoria/23-01686-0034/2023 (H5N3)	PP549216 A/emu/Victoria/21-03712/2021(H10N3)	98.23%
		PP549221 A/wild waterfowl/Western Australia/AS-22-0673-0006/2022(H10N3)	98.16%
		OL370601 A/grey teal/South Australia/18-2812337-47/2018(H1N3)	97.87%
		OL370585 A/grey teal/South Australia/17-3278836-7/2017(H9N3)	97.59%
		OL371081 A/wild duck/Queensland/P20-03375-0084-01/2020(H1N3)	97.45%
	A/Pacific Black Duck/Victoria/23-01686-0039/2023 (H5N3)	PP549216 A/emu/Victoria/21-03712/2021(H10N3)	98.58%
		PP549221 A/wild waterfowl/Western Australia/AS-22-0673-0006/2022(H10N3)	98.51%
		OL370601 A/grey teal/South Australia/18-2812337-47/2018(H1N3)	98.23%
		OL370585 A/grey teal/South Australia/17-3278836-7/2017(H9N3)	97.94%
		OL371081 A/wild duck/Queensland/P20-03375-0084-01/2020(H1N3)	97.80%
	A/Grey Teal/Victoria/23-01688-0045/2023 (mixed)	PP549216 A/emu/Victoria/21-03712/2021(H10N3)	98.23%
		PP549221 A/wild waterfowl/Western Australia/AS-22-0673-0006/2022(H10N3)	98.16%
		OL370601 A/grey teal/South Australia/18-2812337-47/2018(H1N3)	97.87%
		OL370585 A/grey teal/South Australia/17-3278836-7/2017(H9N3)	97.59%
		OL371081 A/wild duck/Queensland/P20-03375-0084-01/2020(H1N3)	97.45%
	A/Pacific Black Duck/Victoria/17363/2023 (H5N3)	PP549216 A/emu/Victoria/21-03712/2021(H10N3)	98.23%
		PP549221 A/wild waterfowl/Western Australia/AS-22-0673-0006/2022(H10N3)	98.16%
		OL370601 A/grey teal/South Australia/18-2812337-47/2018(H1N3)	97.87%
		OL370585 A/grey teal/South Australia/17-3278836-7/2017(H9N3)	97.59%
		OL371081 A/wild duck/Queensland/P20-03375-0084-01/2020(H1N3)	97.45%

NA Subtype	Designation	Top blast hit	% identity
	A/wild waterbird/Queensland/ P23-02457-48/2023(H5N3)	PP549216 A/emu/Victoria/21-03712/2021(H10N3)	98.30%
		PP549221 A/wild waterfowl/Western Australia/AS-22-0673-0006/2022(H10N3)	98.23%
		OL370601 A/grey teal/South Australia/18-2812337-47/2018(H1N3)	97.94%
		OL370585 A/grey teal/South Australia/17-3278836-7/2017(H9N3)	97.66%
		OL371081 A/wild duck/Queensland/P20-03375-0084-01/2020(H1N3)	97.52%
	A/wild waterbird/Queensland/ P23-02457-51/2023 (H5N3)	PP549216 A/emu/Victoria/21-03712/2021(H10N3)	98.30%
		PP549221 A/wild waterfowl/Western Australia/AS-22-0673-0006/2022(H10N3)	98.23%
		OL370601 A/grey teal/South Australia/18-2812337-47/2018(H1N3)	97.94%
		OL370585 A/grey teal/South Australia/17-3278836-7/2017(H9N3)	97.66%
		OL371081 A/wild duck/Queensland/P20-03375-0084-01/2020(H1N3)	97.52%
N7	A/Grey Teal/Victoria/23-01688-0045/2023 (mixed)	OL370323 A/wild duck/New South Wales/M19-11593-66/2019(H7N7)	97.88%
		PP549421 A/chicken/New South Wales/M21-10080-0006/2021(H10N7)	97.53%
		OL370529 A/Pacific black duck/Victoria/18-01589-232/2018(H7N7)	97.53%
		PP549261 A/Grey teal/South Australia/22-64233564-17/2022(H10N7)	96.96%
		PP549293 A/Grey teal/Victoria/15848/2022(H10N7)	96.82%
N9	A/wild waterbird/South Australia/23-80999145-13/2023(H5N9)	OR681022 A/Wild waterfowl/Australia/22-04155-0048/2022(H11N9)	99.79%
		OL370420 A/wild waterbird/Queensland/JCU-272/2019(H5N9)	97.81%
		OL370617 A/grey teal/Victoria/10657/2017(H11N9)	97.17%
		OL371681 A/wild waterbird/Victoria/19-00581-009/2019(H11N9)	97.10%
		OL370448 A/Pacific black duck/Victoria/11889/2019(H11N9)	97.03%

Appendix Table 2. Top 5 Blast hits for all internal segments against the NCBI nt database (September 2024)

Segment	Designation	Top blast hit	% identity
PB2	A/Chestnut Teal/Victoria/23-01686-0034/2023 (H5N3)	PP549211 A/emu/Victoria/21-03712/2021(H10N3)	98.77%
		PP549416 A/chicken/New South Wales/M21-10080-0006/2021(H10N7)	98.73%
		PP549264 A/Grey teal/South Australia/22-64233564-20/2022(H4N6)	98.64%
		OQ201826 A/Wild waterfowl/Australia/22-02927-0052/2022(H4N6)	98.46%
		PP549400 A/Tawny frogmouth/Western Australia/AS-21-1823-0007/2021(H10N7)	98.38%
	A/Pacific Black Duck/Victoria/23-01686-0039/2023 (H5N3)	PP549264 A/Grey teal/South Australia/22-64233564-20/2022(H4N6)	99.12%
		PP549416 A/chicken/New South Wales/M21-10080-0006/2021(H10N7)	98.33%
		PP549211 A/emu/Victoria/21-03712/2021(H10N3)	98.29%
		OQ201826 A/Wild waterfowl/Australia/22-02927-0052/2022(H4N6)	98.07%
		PP549400 A/Tawny frogmouth/Western Australia/AS-21-1823-0007/2021(H10N7)	97.98%
	A/Grey Teal/Victoria/23-01688-0045/2023 (mixed)	PP549264 A/Grey teal/South Australia/22-64233564-20/2022(H4N6)	99.21%
		PP549416 A/chicken/New South Wales/M21-10080-0006/2021(H10N7)	98.42%
		PP549211 A/emu/Victoria/21-03712/2021(H10N3)	98.38%
		OQ201826 A/Wild waterfowl/Australia/22-02927-0052/2022(H4N6)	98.16%

Segment	Designation	Top blast hit	% identity
		PP549400 A/Tawny frogmouth/Western Australia/AS-21-1823-0007/2021(H10N7)	98.07%
	A/Pacific Black Duck/Victoria/17363/2023 (H5N3)	PP549416 A/chicken/New South Wales/M21-10080-0006/2021(H10N7)	98.55%
		PP549211 A/emu/Victoria/21-03712/2021(H10N3)	98.51%
		PP549264 A/Grey teal/South Australia/22-64233564-20/2022(H4N6)	98.46%
		OQ201826 A/Wild waterfowl/Australia/22-02927-0052/2022(H4N6)	98.29%
		ON505898 A/Mallard(Anas platyrhynchos)/South Korea/KNU2021-49/2021(H7N7)	98.25%
	A/wild waterbird/Queensland/ P23-02457-48/2023(H5N3)	PP549240 A/Chestnut teal/Victoria/16148/2022(H10N7)	98.90%
		OR681027 A/Wild waterfowl/Australia/22-04155-0048/2022(H11N9)	98.77%
		PP549211 A/emu/Victoria/21-03712/2021(H10N3)	98.64%
		PP549416 A/chicken/New South Wales/M21-10080-0006/2021(H10N7)	98.55%
		OQ201826 A/Wild waterfowl/Australia/22-02927-0052/2022(H4N6)	98.33%
	A/wild waterbird/Queensland/ P23-02457-51/2023 (H5N3)	PP549240 A/Chestnut teal/Victoria/16148/2022(H10N7)	98.90%
		OR681027 A/Wild waterfowl/Australia/22-04155-0048/2022(H11N9)	98.77%
		PP549211 A/emu/Victoria/21-03712/2021(H10N3)	98.64%
		PP549416 A/chicken/New South Wales/M21-10080-0006/2021(H10N7)	98.55%
		OQ201826 A/Wild waterfowl/Australia/22-02927-0052/2022(H4N6)	98.33%
	A/wild waterbird/South Australia/23-80999145-13/2023(H5N9)	OR681027 A/Wild waterfowl/Australia/22-04155-0048/2022(H11N9)	99.47%
		PP549240 A/Chestnut teal/Victoria/16148/2022(H10N7)	98.99%
		PP549416 A/chicken/New South Wales/M21-10080-0006/2021(H10N7)	98.68%
		PP549211 A/emu/Victoria/21-03712/2021(H10N3)	98.64%
		OQ201826 A/Wild waterfowl/Australia/22-02927-0052/2022(H4N6)	98.51%
PB1	A/Chestnut Teal/Victoria/23-01686-0034/2023 (H5N3)	OR681026 A/Wild waterfowl/Australia/22-04155-0048/2022(H11N9)	98.11%
		OL370533 A/Pacific black duck/Victoria/19-01749-033/2019(H10N5)	97.93%
		OL371677 A/wild waterbird/Victoria/19-00581-009/2019(H11N9)	97.93%
		OL371661 A/wild waterbird/Tasmania/20-2052-11/2020(H1N6)	97.89%
		OL371093 A/wild duck/Queensland/P20-03375-0183-01/2020(H6N1)	97.89%
	A/Pacific Black Duck/Victoria/23-01686-0039/2023 (H5N3)	OL370356 A/wild waterbird/South Australia/19-9013889-61/2019(mixed)	98.77%
		OL370343 A/wild waterbird/South Australia/19-9013889-13/2019(mixed)	98.77%
		OL371565 A/wild waterbird/South Australia/19-9013889-31/2019(H7N1)	98.68%
		OL371589 A/wild waterbird/South Australia/19-9013889-43/2019(H7N1)	98.68%
		OL371485 A/wild waterbird/South Australia/17-6661506-14/2017(H7N5)	97.98%
	A/Grey Teal/Victoria/23-01688-0045/2023 (mixed)	OL370356 A/wild waterbird/South Australia/19-9013889-61/2019(mixed)	98.55%
		OL370343 A/wild waterbird/South Australia/19-9013889-13/2019(mixed)	98.55%
		OL371565 A/wild waterbird/South Australia/19-9013889-31/2019(H7N1)	98.46%
		OL371589 A/wild waterbird/South Australia/19-9013889-43/2019(H7N1)	98.46%
		OL371485 A/wild waterbird/South Australia/17-6661506-14/2017(H7N5)	97.85%
	A/Pacific Black Duck/Victoria/17363/2023 (H5N3)	OL370356 A/wild waterbird/South Australia/19-9013889-61/2019(mixed)	98.55%

Segment	Designation	Top blast hit	% identity
		OL370343 A/wild waterbird/South Australia/19-9013889-13/2019(mixed)	98.55%
		OL371565 A/wild waterbird/South Australia/19-9013889-31/2019(H7N1)	98.46%
		OL371589 A/wild waterbird/South Australia/19-9013889-43/2019(H7N1)	98.46%
		OL371485 A/wild waterbird/South Australia/17-6661506-14/2017(H7N5)	97.85%
	A/wild waterbird/Queensland/ P23-02457-48/2023(H5N3)	PP549241 A/Chestnut teal/Victoria/16148/2022(H10N7)	99.08%
		OQ201827 A/Wild waterfowl/Australia/22-02927-0052/2022(H4N6)	98.90%
		PP549417 A/chicken/New South Wales/M21-10080-0006/2021(H10N7)	98.72%
		ON495895 A/Bean Goose(Anser fabalis)/South Korea/KNU2021-42/2021(H1N1)	98.42%
		OK217082 A/Wild Duck/South Korea/KNU2020-11/2020(H4N6)	98.28%
	A/wild waterbird/Queensland/ P23-02457-51/2023 (H5N3)	PP549241 A/Chestnut teal/Victoria/16148/2022(H10N7)	99.08%
		OQ201827 A/Wild waterfowl/Australia/22-02927-0052/2022(H4N6)	98.90%
		PP549417 A/chicken/New South Wales/M21-10080-0006/2021(H10N7)	98.72%
		ON495895 A/Bean Goose(Anser fabalis)/South Korea/KNU2021-42/2021(H1N1)	98.42%
		OK217082 A/Wild Duck/South Korea/KNU2020-11/2020(H4N6)	98.28%
	A/wild waterbird/South Australia/23-80999145-13/2023(H5N9)	PP549241 A/Chestnut teal/Victoria/16148/2022(H10N7)	99.08%
		OQ201827 A/Wild waterfowl/Australia/22-02927-0052/2022(H4N6)	98.90%
		PP549417 A/chicken/New South Wales/M21-10080-0006/2021(H10N7)	98.72%
		ON495895 A/Bean Goose(Anser fabalis)/South Korea/KNU2021-42/2021(H1N1)	98.42%
		OK217082 A/Wild Duck/South Korea/KNU2020-11/2020(H4N6)	98.28%
PA	A/Chestnut Teal/Victoria/23-01686-0034/2023 (H5N3)	PP549274 A/Grey teal/South Australia/22-64233564-37/2022(H10N2)	99.12%
		PP549258 A/Grey teal/South Australia/22-64233564-17/2022(H10N7)	99.02%
		OL371678 A/wild waterbird/Victoria/19-00581-009/2019(H11N9)	98.51%
		PP549250 A/Grey teal/New South Wales/14340X/2020(H4N6)	98.42%
		OL370510 A/Pacific black duck/Victoria/11840/2019(H2N1)	98.33%
	A/Pacific Black Duck/Victoria/23-01686-0039/2023 (H5N3)	PP549274 A/Grey teal/South Australia/22-64233564-37/2022(H10N2)	98.65%
		PP549258 A/Grey teal/South Australia/22-64233564-17/2022(H10N7)	98.56%
		OL371678 A/wild waterbird/Victoria/19-00581-009/2019(H11N9)	98.33%
		OL370510 A/Pacific black duck/Victoria/11840/2019(H2N1)	98.14%
		PP549250 A/Grey teal/New South Wales/14340X/2020(H4N6)	98.09%
	A/Grey Teal/Victoria/23-01688-0045/2023 (mixed)	PP549274 A/Grey teal/South Australia/22-64233564-37/2022(H10N2)	98.65%
		PP549258 A/Grey teal/South Australia/22-64233564-17/2022(H10N7)	98.56%
		OL371678 A/wild waterbird/Victoria/19-00581-009/2019(H11N9)	98.33%
		OL370510 A/Pacific black duck/Victoria/11840/2019(H2N1)	98.14%
		PP549250 A/Grey teal/New South Wales/14340X/2020(H4N6)	98.09%
	A/Pacific Black Duck/Victoria/17363/2023 (H5N3)	PP549274 A/Grey teal/South Australia/22-64233564-37/2022(H10N2)	98.61%
		PP549258 A/Grey teal/South Australia/22-64233564-17/2022(H10N7)	98.51%
		OL371678 A/wild waterbird/Victoria/19-00581-009/2019(H11N9)	98.19%

Segment	Designation	Top blast hit	% identity
		OL370510 A/Pacific black duck/Victoria/11840/2019(H2N1)	98.00%
		PP549250 A/Grey teal/New South Wales/14340X/2020(H4N6)	98.00%
	A/wild waterbird/Queensland/ P23-02457-48/2023(H5N3)	PP549274 A/Grey teal/South Australia/22-64233564-37/2022(H10N2)	98.79%
		PP549258 A/Grey teal/South Australia/22-64233564-17/2022(H10N7)	98.70%
		OL371678 A/wild waterbird/Victoria/19-00581-009/2019(H11N9)	98.09%
		PP549250 A/Grey teal/New South Wales/14340X/2020(H4N6)	98.09%
		OL370445 A/Pacific black duck/Victoria/11889/2019(H11N9)	97.95%
	A/wild waterbird/Queensland/ P23-02457-51/2023 (H5N3)	PP549274 A/Grey teal/South Australia/22-64233564-37/2022(H10N2)	98.79%
		PP549258 A/Grey teal/South Australia/22-64233564-17/2022(H10N7)	98.70%
		OL371678 A/wild waterbird/Victoria/19-00581-009/2019(H11N9)	98.09%
		PP549250 A/Grey teal/New South Wales/14340X/2020(H4N6)	98.09%
		OL370445 A/Pacific black duck/Victoria/11889/2019(H11N9)	97.95%
	A/wild waterbird/South Australia/23-80999145-13/2023(H5N9)	PP549274 A/Grey teal/South Australia/22-64233564-37/2022(H10N2)	98.84%
		PP549258 A/Grey teal/South Australia/22-64233564-17/2022(H10N7)	98.74%
		OL371678 A/wild waterbird/Victoria/19-00581-009/2019(H11N9)	98.14%
		PP549250 A/Grey teal/New South Wales/14340X/2020(H4N6)	98.14%
		OL370445 A/Pacific black duck/Victoria/11889/2019(H11N9)	98.09%
NP	A/Radjah Shelduck/Northern Territory/ 20231282-03/2023(H5N1)	OL371920 A/wild waterbird/Western Australia/AS19-3999-1/2019(H8N4)	98.20%
		OL372192 A/wild waterfowl/Western Australia/AS18-1331-10/2018(H11N3)	97.66%
		OL370274 A/wild waterfowl/Western Australia/AS18-1331-84/2018(H11N9)	97.60%
		OL370267 A/wild waterfowl/Western Australia/AS18-1331-79/2018(H11N9)	97.53%
		OL370512 A/Pacific black duck/Victoria/11840/2019(H2N1)	97.46%
	A/Chestnut Teal/Victoria/23-01686-0034/2023 (H5N3)	OL370413 A/wild duck/Queensland/P20-00339-68/2020(mixed)	98.40%
		PP549236 A/Chestnut teal/Victoria/16099/2022(H10N7)	98.20%
		OL371680 A/wild waterbird/Victoria/19-00581-009/2019(H11N9)	98.20%
		OL370600 A/grey teal/South Australia/18-2812337-47/2018(H1N3)	98.20%
		PP549228 A/Chestnut teal/Victoria/16047/2022(H10N7)	98.13%
	A/Pacific Black Duck/Victoria/23-01686-0039/2023 (H5N3)	PP549236 A/Chestnut teal/Victoria/16099/2022(H10N7)	98.73%
		PP549228 A/Chestnut teal/Victoria/16047/2022(H10N7)	98.66%
		OL370413 A/wild duck/Queensland/P20-00339-68/2020(mixed)	98.60%
		OL371680 A/wild waterbird/Victoria/19-00581-009/2019(H11N9)	98.53%
		OL370600 A/grey teal/South Australia/18-2812337-47/2018(H1N3)	98.53%
	A/Grey Teal/Victoria/23-01688-0045/2023 (mixed)	PP549236 A/Chestnut teal/Victoria/16099/2022(H10N7)	98.73%
		PP549228 A/Chestnut teal/Victoria/16047/2022(H10N7)	98.66%
		OL370413 A/wild duck/Queensland/P20-00339-68/2020(mixed)	98.60%
		OL371680 A/wild waterbird/Victoria/19-00581-009/2019(H11N9)	98.53%
		OL370600 A/grey teal/South Australia/18-2812337-47/2018(H1N3)	98.53%
	A/Pacific Black Duck/Victoria/17363/2023 (H5N3)	PP549260 A/Grey teal/South Australia/22-64233564-17/2022(H10N7)	98.60%
		PP549348 A/Pacific black duck/Tasmania/22-1184-5/2022(H4N1)	98.20%

Segment	Designation	Top blast hit	% identity
		MN826602 A/Chestnut teal/Australia/CT08.18-12952/2018(H9N2)	98.06%
		OL370447 A/Pacific black duck/Victoria/11889/2019(H11N9)	98.00%
		OL370600 A/grey teal/South Australia/18-2812337-47/2018(H1N3)	98.00%
	A/wild waterbird/Queensland/ P23-02457-48/2023(H5N3)	OR681023 A/Wild waterfowl/Australia/22-04155-0048/2022(H11N9)	99.00%
		OL370413 A/wild duck/Queensland/P20-00339-68/2020(mixed)	98.26%
		OL371680 A/wild waterbird/Victoria/19-00581-009/2019(H11N9)	98.20%
		OL370600 A/grey teal/South Australia/18-2812337-47/2018(H1N3)	98.20%
		OL370328 A/wild waterbird/South Australia/19-6528228-82/2019(H11)	98.13%
	A/wild waterbird/Queensland/ P23-02457-51/2023 (H5N3)	OR681023 A/Wild waterfowl/Australia/22-04155-0048/2022(H11N9)	99.00%
		OL370413 A/wild duck/Queensland/P20-00339-68/2020(mixed)	98.26%
		OL371680 A/wild waterbird/Victoria/19-00581-009/2019(H11N9)	98.20%
		OL370600 A/grey teal/South Australia/18-2812337-47/2018(H1N3)	98.20%
		OL370328 A/wild waterbird/South Australia/19-6528228-82/2019(H11)	98.13%
	A/wild waterbird/South Australia/23-80999145-13/2023(H5N9)	OL370944 A/wild bird/Victoria/20-03245-0029/2020(H9N2)	98.46%
		OL371080 A/wild duck/Queensland/P20-03375-0084-01/2020(H1N3)	98.46%
		OL371104 A/wild duck/Queensland/P20-03558-0029-01/2020(H1N3)	98.40%
		OL370367 A/wild waterbird/South Australia/19-9013889-76/2019(H7N1)	98.20%
		OL371600 A/wild waterbird/South Australia/19-9013889-45/2019(H7N1)	98.13%
M	A/Radjah Shelduck/Northern Territory/ 20231282-03/2023(H5N1)	OQ201832 A/Wild waterfowl/Australia/22-02927-0052/2022(H4N6)	99.80%
		MW466354 A/duck/Bangladesh/41797/2019(H3N8)	99.80%
		OR949087 A/duck/Chiayi/19WB0021F/2018(H4N6)	99.80%
		ON505895 A/Mallard(Anas platyrhynchos)/South Korea/KNU2021-41/2021(H9N2)	99.80%
		OK235643 A/Wild Duck/South Korea/KNU2020-74/2020(H3N8)	99.80%
	A/Chestnut Teal/Victoria/23-01686-0034/2023 (H5N3)	OQ201832 A/Wild waterfowl/Australia/22-02927-0052/2022(H4N6)	99.80%
		MW466354 A/duck/Bangladesh/41797/2019(H3N8)	99.49%
		OR758553 A/chicken/Dhaka/BAIV-1050_M/2021(H9N2)	99.49%
		OR949087 A/duck/Chiayi/19WB0021F/2018(H4N6)	99.49%
		ON505895 A/Mallard(Anas platyrhynchos)/South Korea/KNU2021-41/2021(H9N2)	99.49%
	A/Pacific Black Duck/Victoria/23-01686-0039/2023 (H5N3)	PP549238 A/Chestnut teal/Victoria/16099/2022(H10N7)	99.29%
		MW466354 A/duck/Bangladesh/41797/2019(H3N8)	99.19%
		PP549230 A/Chestnut teal/Victoria/16047/2022(H10N7)	99.19%
		PP549310 A/Grey teal/Victoria/16156/2022(H10N7)	99.19%
		OR949087 A/duck/Chiayi/19WB0021F/2018(H4N6)	99.19%
	A/Grey Teal/Victoria/23-01688-0045/2023 (mixed)	PP549238 A/Chestnut teal/Victoria/16099/2022(H10N7)	99.29%
		MW466354 A/duck/Bangladesh/41797/2019(H3N8)	99.19%
		PP549230 A/Chestnut teal/Victoria/16047/2022(H10N7)	99.19%
		PP549310 A/Grey teal/Victoria/16156/2022(H10N7)	99.19%
		OR949087 A/duck/Chiayi/19WB0021F/2018(H4N6)	99.19%
	A/Pacific Black Duck/Victoria/17363/2023 (H5N3)	PP549238 A/Chestnut teal/Victoria/16099/2022(H10N7)	98.98%
		MW466354 A/duck/Bangladesh/41797/2019(H3N8)	98.88%
		PP549230 A/Chestnut teal/Victoria/16047/2022(H10N7)	98.88%
		PP549310 A/Grey teal/Victoria/16156/2022(H10N7)	98.88%
		OR949087 A/duck/Chiayi/19WB0021F/2018(H4N6)	98.88%
	A/wild waterbird/Queensland/ P23-02457-48/2023(H5N3)	OL370602 A/grey teal/South Australia/18-2812337-47/2018(H1N3)	99.08%
		OL370421 A/wild waterbird/Queensland/JCU-272/2019(H5N9)	98.88%

Segment	Designation	Top blast hit	% identity
		MN826604 A/Chestnut teal/Australia/CT08.18-12952/2018(H9N2)	98.88%
		OL371538 A/wild waterbird/South Australia/19-6529700-80/2019(H8N4)	98.88%
		OL371530 A/wild waterbird/South Australia/19-6529700-27/2019(H6N8)	98.78%
	A/wild waterbird/Queensland/ P23-02457-51/2023 (H5N3)	OL370602 A/grey teal/South Australia/18-2812337-47/2018(H1N3)	99.08%
		OL370421 A/wild waterbird/Queensland/JCU-272/2019(H5N9)	98.88%
		MN826604 A/Chestnut teal/Australia/CT08.18-12952/2018(H9N2)	98.88%
		OL371538 A/wild waterbird/South Australia/19-6529700-80/2019(H8N4)	98.88%
		OL371530 A/wild waterbird/South Australia/19-6529700-27/2019(H6N8)	98.78%
	A/wild waterbird/South Australia/23-80999145-13/2023(H5N9)	OR950095 A/duck/Tainan/16WB3443-16-20/2016(H7N7)	99.49%
		OR675273 A/Spot-billed duck(Anas poecilohyncha)/Korea/KNU27/2023(H11N15)	99.39%
		MW466354 A/duck/Bangladesh/41797/2019(H3N8)	99.39%
		OR949087 A/duck/Chiayi/19WB0021F/2018(H4N6)	99.39%
		OQ201832 A/Wild waterfowl/Australia/22-02927-0052/2022(H4N6)	99.39%
NS	A/Radjah Shelduck/Northern Territory/ 20231282-03/2023(H5N1)	PP549407 A/Tawny frogmouth/Western Australia/AS-21-1823-0007/2021(H10N7)	99.40%
		ON495909 A/Mallard(Anas platyrhynchos)/South Korea/KNU2021-44/2021(H1N1)	99.28%
		MF694254 A/domestic duck/Georgia/11/2016(H4N6)	99.28%
		MN208025 A/teal/Egypt/MB-D-125OP/2015(H7N3)	99.28%
		MF694195 A/domestic duck/Georgia/4/2016(H4N6)	99.28%
	A/Chestnut Teal/Victoria/23-01686-0034/2023 (H5N3)	PP549407 A/Tawny frogmouth/Western Australia/AS-21-1823-0007/2021(H10N7)	99.52%
		ON495909 A/Mallard(Anas platyrhynchos)/South Korea/KNU2021-44/2021(H1N1)	99.40%
		MF694254 A/domestic duck/Georgia/11/2016(H4N6)	99.40%
		MN208025 A/teal/Egypt/MB-D-125OP/2015(H7N3)	99.40%
		MF694195 A/domestic duck/Georgia/4/2016(H4N6)	99.40%
	A/Pacific Black Duck/Victoria/23-01686-0039/2023 (H5N3)	PP549210 A/wild waterbird/South Australia/22-68204541-55/2022(H4N6)	99.52%
		PP549231 A/Chestnut teal/Victoria/16047/2022(H10N7)	99.40%
		OL370450 A/Pacific black duck/Victoria/11889/2019(H11N9)	99.40%
		PP549311 A/Grey teal/Victoria/16156/2022(H10N7)	99.28%
		OR681024 A/Wild waterfowl/Australia/22-04155-0048/2022(H11N9)	99.16%
	A/Grey Teal/Victoria/23-01688-0045/2023 (mixed)	PP549210 A/wild waterbird/South Australia/22-68204541-55/2022(H4N6)	99.52%
		PP549231 A/Chestnut teal/Victoria/16047/2022(H10N7)	99.40%
		OL370450 A/Pacific black duck/Victoria/11889/2019(H11N9)	99.40%
		PP549311 A/Grey teal/Victoria/16156/2022(H10N7)	99.28%
		OR681024 A/Wild waterfowl/Australia/22-04155-0048/2022(H11N9)	99.16%
	A/Pacific Black Duck/Victoria/17363/2023 (H5N3)	PP549407 A/Tawny frogmouth/Western Australia/AS-21-1823-0007/2021(H10N7)	99.16%
		ON495909 A/Mallard(Anas platyrhynchos)/South Korea/KNU2021-44/2021(H1N1)	99.05%
		MF694254 A/domestic duck/Georgia/11/2016(H4N6)	99.05%
		MN208025 A/teal/Egypt/MB-D-125OP/2015(H7N3)	99.05%
		MF694195 A/domestic duck/Georgia/4/2016(H4N6)	99.05%
	A/wild waterbird/Queensland/ P23-02457-48/2023(H5N3)	PP549407 A/Tawny frogmouth/Western Australia/AS-21-1823-0007/2021(H10N7)	99.76%
		ON495909 A/Mallard(Anas platyrhynchos)/South Korea/KNU2021-44/2021(H1N1)	99.64%
		MF694254 A/domestic duck/Georgia/11/2016(H4N6)	99.64%
		MN208025 A/teal/Egypt/MB-D-125OP/2015(H7N3)	99.64%
		MF694195 A/domestic duck/Georgia/4/2016(H4N6)	99.52%
	A/wild waterbird/Queensland/ P23-02457-51/2023 (H5N3)	PP549407 A/Tawny frogmouth/Western Australia/AS-21-1823-0007/2021(H10N7)	99.76%

Segment	Designation	Top blast hit	% identity
	A/wild waterbird/South Australia/23-80999145-13/2023(H5N9)	ON495909 A/Mallard(Anas platyrhynchos)/South Korea/KNU2021-44/2021(H1N1)	99.64%
		MF694254 A/domestic duck/Georgia/11/2016(H4N6)	99.64%
		MN208025 A/teal/Egypt/MB-D-125OP/2015(H7N3)	99.64%
		MF694195 A/domestic duck/Georgia/4/2016(H4N6)	99.52%
		OR681024 A/Wild waterfowl/Australia/22-04155-0048/2022(H11N9))	99.76%
		PP549210 A/wild waterbird/South Australia/22-68204541-55/2022(H4N6)	99.64%
		PP549231 A/Chestnut teal/Victoria/16047/2022(H10N7)	99.52%
		OL370450 A/Pacific black duck/Victoria/11889/2019(H11N9)	99.52%
		PP549311 A/Grey teal/Victoria/16156/2022(H10N7)	99.40%

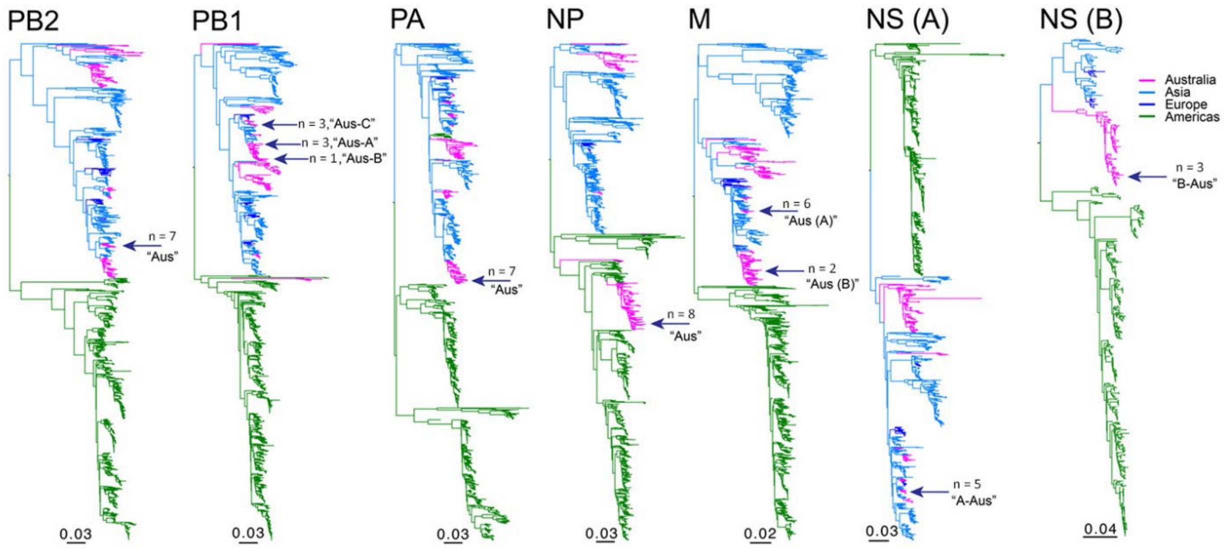

Appendix Figure. Maximum likelihood trees for internal segments. In cases where all viruses are similar only a single arrow is shown. In cases where viruses fall into different Australian lineages (i.e. a result of different virus incursion events). Lineage names presented in Table 1 and the number of sequences in each of those lineages have been included adjacent to arrows. Lineage names are for clarity only. No internal segments are available for either virus from Queensland, and PB1, PB2 and PA segments were unavailable for A/Radjah shelduck/Northern Territory/20231282-03/2023(H5N1). Alignments for all trees are available on https://github.com/michellewille2/Eurasian-LPAI-H5-incursion-to-Australia
